# A quantitative evaluation of empathy using JSE-S Tool, before and after a Medical Humanities Module, amongst first-year medical students in Nepal

**DOI:** 10.1186/s12909-022-03188-y

**Published:** 2022-03-08

**Authors:** Krishna Bahadur G. C., Amit Arjyal, Amanda Helen Douglas, Madhusudan Subedi, Rajesh Gongal

**Affiliations:** 1 Department of Community Health Sciences, Patan Academy of Health Sciences, GPO Box 26500 Ward No.5, Bagmati Province Lalitpur Metropolitan City, Nepal; 2 Department of General Practice and Emergency Medicine, Patan Academy of Health Sciences, Lalitpur, Nepal; 3 Department of Surgery, School of Medicine, Patan Academy of Health Sciences, Lalitpur, Nepal

**Keywords:** Empathy, Medical humanities, Jefferson Scale of Empathy-Student version, JSE-S, Medical education, Undergraduate medical curriculum

## Abstract

**Background:**

Doctors’ empathy: the understanding of patients’ experiences, concerns and perspectives, is highly valued by patients yet often lacking in patient care. Medical Humanities has been introduced within undergraduate curriculum to address this lack in empathy. There is a paucity of research on the impact of a course on medical humanities on the empathy of medical students, particularly in South Asia. Here we report on the impact of such an intervention in first-year medical students and aim to help outcome-based medical education and the evaluation and promotion of humanities within medical courses.

**Methods:**

This study is a quantitative evaluation of student empathy before and after a Medical Humanities Module. The study employs the Jefferson Scale of Empathy-Student version (JSE-S). Participants were first-year medical students at Patan Academy of Health Sciences, Nepal. All cohort students were invited to participate and written consent was obtained. Data were collected both prior-to and on-completion-of, a six-week Medical Humanities Module. Pre- and post-module data were analyzed and the resulting empathy scores compared using the paired t-test or Wilcoxon signed-rank test. Subgroup analysis was undertaken to determine the association of the score with gender and preferred future speciality.

**Results:**

Sixty-two student responses were analyzed, 32 (52%) of whom were male. In the pre-module scores females had a slightly higher mean score than males:108 and 103 respectively. Participants who preferred people-oriented specialities also scored higher than those preferring procedure and technology-oriented specialities: 107 and 103 respectively. There was a significant increase in mean score for the entire class from pre-module to post-module: 105 to 116, p-value of < 0.001. Mean scores rose from 103 to 116 in males, and from 108 to 116 in females. Participants preferring procedure and technology-oriented specialities showed a significant increase in mean scores:103 to 117, and participants preferring people-oriented specialities demonstrated a smaller increase:107 to 111.

**Conclusions:**

This study provides evidence of the impact of a Medical Humanities course for increasing medical student empathy scores at an institution in Nepal. Teaching of Medical Humanities is an important contributor to the development of empathy in medical students and its widespread expansion in the whole of South Asia should be considered.

**Supplementary Information:**

The online version contains supplementary material available at 10.1186/s12909-022-03188-y.

## Background

Empathy is an important quality for medical doctors and highly valued by patients [1]. In the context of patient care, empathy is described as a predominantly cognitive attribute involving an understanding of patients’ experiences, concerns and perspectives, and communicating this, rather than sharing patient feelings [2].

The ability of doctors to empathise, that is, to recognise, relate to and understand another’s emotional situation, is important to patients [3]. Physicians’ empathy has a significant impact on patients’ reporting of symptoms [4], disease outcomes [5], and patient satisfaction and compliance [3]. In the present era of patient-centred care, empathy is a key component of a doctor’s professionalism. Producing graduates with a high degree of empathy should be the goal of all medical educators [6].

Despite this, in recent years a lack of empathy among doctors and decline in communication and emotional support from healthcare professionals has been reported [7, 8]. There are few Nepal-based studies of student empathy, however, one study found lower empathy scores among Nepali final year medical students than those in developed countries [9]. From elsewhere in South Asia-Bangladesh, India and Pakistan, in recent years, there have been several reports on the assessment of empathy in medical students [10–12]. These studies point out low levels of empathy and also stress the need to inculcate empathy by means of a formal curriculum.

The question of whether empathy can be taught is much debated, but there is general indication that empathy may be subject to positive change with a range of interventional strategies [13]. For many years the lack of educational experiences have been identified as a contributor to low empathy amongst medical students [14]. Indeed, traditional medical education often contributes to the problem as students’ empathy and compassion decline during their training [15, 16]. Empathy has been shown to increase following different interventions emphasizing empathy such as integrating early patient contact with communication and interaction teaching [17], patient narrative and creative arts, writing, drama, and experiential learning [18].

Medical Humanities explores human experiences through the media of arts, literature, drama, music, film and philosophy [19]. Medical Humanities courses have been designed and implemented within medical curricula, partially to address the problem of low empathy amongst students [17, 18, 20, 21]. Many medical schools across the world have incorporated Medical Humanities in their undergraduate curricula [19], although these vary greatly in their content. Student empathy has been shown to increase following Medical Humanities teaching [22, 23] and it is likely that Medical Humanities teaching will result in more compassionate doctors [24, 25] although there is currently no evidence to support this [19]. Ultimately, patients are the potential beneficiaries of such Medical Humanities courses [20].

In 2018 Patan Academy of Health Sciences(PAHS) in Lalitpur District of Nepal, commenced a new 16-hour-long Medical Humanities module within the introductory foundation course for first year medical students [26]. The Medical Humanities module explores diverse topics around disability, the elderly, death and dying, social injustice, compassion, and doctor-patient relationship through various media such as art, photography, literature, film and poetry.

Medical Humanities teaching is relatively new to Nepal. In other South Asian countries, the idea of introducing Medical Humanities in the medical curriculum has both been pondered on and tried [27–30]. It is important to evaluate the impact of educational interventions, particularly in new contexts. Evaluation can facilitate the development of outcome-based education and provide evidence to support the expansion of medical humanities education within medical schools in South Asia [31]. PAHS’s Medical Humanities course is positively perceived by learners who found it enjoyable and interesting, and believed it made them think differently and helped them to understand a doctor’s role in caring; they also felt that it would make them better doctors [26]. However, there has been no evaluation of the course’s impact on student empathy.

There is one Nepal-based study measuring the effect of a Medical Humanities course on empathy, using the Interpersonal Reactivity Index (IRI), which showed an increase in the perspective taking component of empathy but was unclear on the overall score impact [32]. There have been no studies in Nepal or South Asia, evaluating student empathy before and after a Medical Humanities course using the Jefferson Scale of physician empathy.

In addition, there are various factors that affect medical students’ empathy score. Gender, ethnic and social background and the speciality that they wish to choose in the future have been shown to be associated with empathy score in studies done on medical students elsewhere. Determining these associations will point out groups to whom empathy education must be emphasized.

This study evaluates the empathy levels of a first year cohort of medical students, before and after undertaking a Medical Humanities Module. This is the first report on observations being done on a longitudinal cohort of medical students of admission year 2019-20 at Patan Academy of Health Sciences.

## Methods

### Setting and participants

This study was carried out amongst first year medical students(Bachelor of Medicine and Bachelor of Surgery-MBBS students) admitted to Patan Academy of Health Sciences(PAHS) in the academic year 2019-20. PAHS is a public, not-for-profit, tertiary academic institution dedicated to improving Nepal’s rural health by training health workers. It also aims to serve as a model of innovative medical education in a developing country, which can be replicated elsewhere. PAHS is located in the city of Lalitpur, adjacent to the capital of Nepal, Kathmandu and is based at Patan Hospital which is the major teaching hospital for the academy. Admission to PAHS takes place after a nationwide common medical entrance examination. All 65 students in the admission year 2019-20 were invited to participate in this study. All the students in the academic year group who consented to participate were eligible for inclusion. To minimize the effect of coercion, unquestioned freedom to refuse to participate was emphasized to all the students.

### Study instrument

This study utilized the Jefferson Scale of Empathy-Student Version (JSE-S), a self-administered written questionnaire, developed to measure empathetic qualities and tendencies amongst healthcare students and professionals [33]. The JSE-S is a validated, 20-statement item questionnaire with participant responses scored on a seven-point Likert scale. Ten positively worded items, linked to “perspective-taking” were scored directly (strongly disagree = 1, strongly agree = 7), whilst 10 negatively worded statement items were reverse scored ( strongly disagree = 7, strongly agree = 1). Eight of the negative statement items were regarding “compassionate care” and two concerned “standing in the patient’s shoes”. The total scores were calculated with a possible range of 20 to 140, higher values indicating a greater degree of empathy [34].

Information related to student demographics and their preferred future medical speciality, was also collected.

### Study procedures

The Bachelor of Medicine, Bachelor of Surgery (MBBS) course at Patan Academy of Health Sciences begins in the first year with an eight-week-long Foundation Block. This study was carried out during the Foundation Block. The Foundation Block has a 16-hour-long Medical Humanities Module, which begins in the first week and ends in the last week of the block. The course employs various media such as: art, photography, literature, film and poetry. Stimulus material is provided and students engaged in active learning through small group discussion, presentations, poster design and drama [26]. Students undertake a disability exposure: inhabiting the roles of carers and physically disabled people, they visit the local area. After the activity students reflect on their experience. At the end of the course students produce individual written reflections and group dramas, exploring their experiences and learning. A detailed description of selected contents of the Medical Humanities Module at PAHS is provided in Sect. 1 of the Supplementary Appendix.

All students in the admission year 2019-20 were invited to participate. As the study was being carried out by faculty members, in order to reduce the risk of bias and the effect of coercion, all the students were clearly explained that they were free to deny consent to participate and no further questions would be asked of them for doing so. The questionnaire was anonymized to reduce response or conformity bias. The investigators (KGC and AA) who administered and analysed the JSE-S tool were not involved in designing or teaching the Medical Humanities Course.

Potential participants were approached by the researchers as a cohort, prior to the start of the module (Pre-module Assessment) and again upon its completion (Post-module Assessment). Data were collected through participants written completion of the JSE-S tool. The study was conducted in English, the language of medical education in Nepal.

### Study definitions

The ‘Future Speciality’ was defined as the medical speciality the first-year students wished to pursue after graduation or the career path they were interested in at the time of this survey. Future Speciality is categorized into three broad groups: ‘People-Oriented’ which includes internal medicine, pediatrics, obstetrics-gynecology, family medicine, and psychiatry; ‘Technology and Procedure-Oriented’ which includes surgery, neurosurgery, orthopedic surgery, and public health; and ‘Undecided’, for students unable to identify a future speciality they would be interested in pursuing later in their careers. Specialities that require frequent and continuous encounters with patients and preventive care consultations are grouped in the ‘People-Oriented’ specialities and specialities that require more technical and procedural skills are grouped in the ‘Technology and Procedure-Oriented’ specialities. This broad grouping of speciality classification has been used in previous empathy studies [35].

### Data and statistical procedures

After collection of completed survey tools, data was entered into Microsoft excel and checked for discrepancies. Data was double-checked for duplicates and outliers, which were removed as necessary.

Prior to analysis, the scalar data were scored or reverse-scored as necessary, for the positively and negatively worded items respectively, based on the JSE-S Professional Manual and Users Guide (2009) [36]. Statistical analysis was conducted based on simple descriptive statistics using grouped mean scores, as outlined in the JSE-S methodology. The empathy scores were compared for the whole cohort and subgroups, using, independent T-test and ANOVA test respectively.

Similarly, to compare the significance of pre-module and post-module score difference, paired t-test was applied depending on the normality of data. For non-normally distributed data the nonparametric test Wilcoxon signed-rank test was applied. Tests were considered statistically significant at p value of ≤ 0.05 level. All analysis was conducted using the statistical software package SPSS 20.

### Ethical consideration

Participation in the study was voluntary and students understood that they were free to decline to participate or withdraw from the study, with no negative consequences. The purpose of the study was explained to potential participants. Participants understood that their identity would be kept confidential and that the data would be used for research and potentially published. Informed voluntary written consent was obtained from the participants. Participants’ responses were anonymized in the data input process, through allocation of a code to each participant. Participant responses and data were kept securely and accessible only to the research team. The study proposal received local ethical approval from the Institutional Review Committee at PAHS.

## Results

All 65 students in the study cohort consented to participate. Data from three students were removed from the final analysis due to extremely low and discrepant scores. The final data analysis was carried out on 62 students’ responses. Thirty-two (51.6%) students were male and 30(48.4%)female. Sixty(96.8%) students were younger than 22 years of age.

Table 1 shows students’ preference of Future Speciality(1a) and their preference according to speciality group: People-Oriented speciality or Technology and Procedure-Oriented speciality(1b). Surgery and neurosurgery were the most popular specialities. The majority of students chose a Technology and Procedure-Oriented speciality.

**Table 1:** Distribution of participants by Future Speciality

	Frequency (*n* = 62)	Percentage
**1a Future Speciality**		
Surgery	18	29.0
Neurosurgery	12	19.4
Internal Medicine	4	6.5
Pediatrics	2	3.2
Psychiatry	2	3.2
Neurology	2	3.2
Orthopedic Surgery	2	3.2
Obstetrics/Gynecology	1	1.6
Family Medicine/General Practice	1	1.6
Preventive Medicine	1	1.6
Public Health	1	1.6
Undecided	16	25.8
**1b Future Speciality Group**		
People-Oriented	13	21.0
Technology and Procedure-Oriented	33	53.2
Undecided	16	25.8

Cronbach’s alpha was computed for both Pre and Post-module Assessment, and found to be 0.703 and 0.629 respectively.

In the gender-based comparison of scores, mean(± SD) empathy score was slightly different for males, 103.13(± 10.24), versus females, 108.07(± 10.22) in the Pre-module Assessment. Female students had a slightly higher pre-module mean score, however, this difference was not statistically significant, (*p*-value = 0.062). The Post-module Assessment revealed no difference between the sexes, with mean(± SD) empathy score of 116.44(± 8.94) for males and 116.13(± 9.25) for females (*p*-value = 0.896).

Table 2 shows a similar comparison between the Pre and Post-module scores based on Future Speciality. The mean(± SD)Pre-module scores were slightly lower amongst the Procedure-oriented specialities group, however, this difference was not statistically significant. Likewise, Post-module scores analysis revealed no statistically significant difference.

**Table 2:** Comparison of Future Speciality group scores, Pre- and Post-module

Jefferson Scale of Empathy Timing of Assessment	Subgroup of Future Speciality			*p-*value*
	People Oriented [Mean ± SD]	Procedure or Technology Oriented [Mean ± SD]	Undecided [Mean ± SD]	
Pre-module	107.31 ± 9.54	103.12 ± 9.84	109 ± 11.66	0.143
Post-module	111.54 ± 12.14	117.45 ± 8.52	117.75 ± 5.74	0.100

*ANOVA test applied

Table 3 demonstrates a statistically significant (*p* value < 0.001) change in mean empathy score for the entire class cohort from, Pre-module score 105.52(± 10.45) to Post-module score 116.29(± 9.02). This increase was seen for both males and females.

**Table 3:** Comparison of Pre-module versus Post-module scores overall and by gender

Gender	Pre-module Score [Mean ± SD]	Post-module Score [Mean ± SD]	*p-*value*
Entire Class (*n* = 62)	105.52 ± 10.45	116.29 ± 9.02	< 0.001
Males (*n* = 32)	103.13 ± 10.24	116.44 ± 8.94	< 0.001
Females (*n* = 30)	108.07 ± 10.22	116.13 ± 9.25	< 0.001

*Paired t-test for score comparison

In Table 4 Pre- and Post-module scores are compared according to future speciality groups. This demonstrates an increase in scores for all groups, however this difference is highly significant for the Technology and Procedure group, with the mean rising 14.3 points, from 103.12 to 117.45 (*p*-value < 0.001), and Undecided group mean increasing 8.75 points (*p* = 0.0.017).

**Table 4:** Comparison of Pre-module versus Post-module scores in each Future Speciality group

Future Speciality Group	Pre-module Score [Mean ± SD]	Post-module Score [Mean ± SD]	*p-*value*
People Oriented (*n* = 13)	107.31 ± 9.54	111.54 ± 12.14	0.096
Procedure/Technology Oriented (*n* = 33)	103.12 ± 9.84	117.45 ± 8.52	< 0.001
Undecided (*n* = 16)	109 ± 11.66	117.75 ± 5.74	0.017

*Paired t-test for score comparison

The change in Pre and Post-module scores for each sub-section of the Jefferson Scale was calculated (Table 5) and shows a statistically significant increase in Perspective Taking scores, 59.8 to 61.8, (*p*-value = 0.001), and the Compassionate Care scores, 43.5 to 47.0, (*p*-value = 0.005). However, mean scores for the Standing on Patients Shoes sub-section declined slightly, although not statistically significantly (*p* = 0.054).

**Table 5:** Comparison of Pre-module versus Post-module scores in each JSE item subgroup

Jefferson Scale of Empathy Item Subgroup	Score Range	Pre-module Score	Post-module Score	*p*-value
Perspective Taking Score [Mean ± SD]	10–70	59.81 ± 6.42	61.84 ± 5.62	0.0013^a^
Compassionate Care Score [Median(IQR)]#	8–56	43.5 (40.75, 48)	47 (43.75, 49)	0.005^b^
Standing on Patients’ Shoes Score [Mean ± SD]	2–14	8.74 ± 2.37	8.1 ± 2.73	0.054^a^

^a^Paired t-test used to test for significance of difference between Pre-module and Post-module scores

^b^Median was computed for this score as it was non-normally distributed and Wilcoxon signed-rank test was applied for testing significance of difference between pre-module and post-module score

**Fig. 1:**
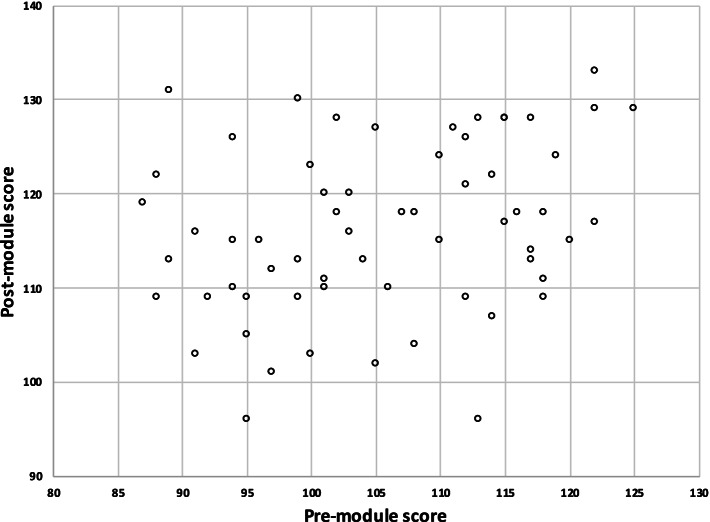
Graph showing correlation between Pre-module and Post-module Scores *(The Pearson’s Correlation Coefficient between the two scores was 0.29 with a p value of 0.02.)*

Correlation of Pre and post-module scores was observed at the individual level with Pearson’s Correlation Coefficient of 0.29(*p* value=0.02). (Fig. 1).

Aggregating the findings in all the students revealed an increase in the mean scores from Pre-module score of 105 to a Post-module score of 116.

## Discussion

This study carried out on first year MBBS students at Patan Academy of Health Sciences measured the scores obtained in the Jefferson Scale of Empathy before and after a module on Medical Humanities. There was an increase in the aggregate mean scores from Pre-module score of 105 to a Post-module score of 116.

This study found increased Post-module empathy scores across all speciality preference groups and both genders.

Our results also concur with those of previous studies regarding the association of empathy with gender and speciality preference. The finding of higher Pre-module empathy scores for female students, although not statistically significant in our study, was similar to that seen in a large study based in the United States of America amongst osteopathic medicine students [35].

Our findings of higher scores for students preferring People-oriented, compared to Technology and Procedure-oriented speciality reflect those of other studies [37]. This demonstrates the positive impact of medical humanities teaching to all students. The fact that Technology and Procedure-oriented speciality group had lower pre-module scores but had higher gains could hint that there is a different perception towards medical education and careers of this group compared to the People-oriented group and giving them a education on humanities could have a large impact. This finding should be corroborated in the future by larger studies both in medical students and doctors practicing in the various specialities. Our findings indicate the potential for identifying students with lowest empathy scores, who may benefit most from medical humanities, based on their gender and preferred future speciality.

A similar Nepal-based study also demonstrated improvement in some components of empathy following a medical humanities course, although direct score comparison is not possible due to different instrument usage [32]. Whilst our observed improvement in empathy scores following a Medical Humanities course are encouraging, there is uncertainty regarding its long-term impact. Sustained improvement in empathic understanding has been observed after repeated interventions. A study that had examined the changes in empathy scores after a targeted activity such as watching a videos of patient encounters aimed at enhancing empathetic understanding showed that empathy levels sustainably enhance after targeted and repeated interventions compared to a control group for which there was only a short intervention or no intervention was carried out at all [38] indicating the potential for similar impact amongst our participants given the prolonged duration of the intervention; however, this needs to be evaluated in future studies. Unlike that study, our study did not have a control group for comparison, as our module was deemed to be essential and not optional, but the extensive module that we taught may have been able to enhance empathetic understanding in a similar method as this experimental study.

Studies have also been carried out to assess the change in empathy scores after specific interventions other than a medical humanities module like ours. One study shows the increase in empathy scores after completion of a single session course in communication skills [39]. Patient centred communication has also been shown to be correlated with empathy scores in another study [40].

Our study participants, in the embryonic stages of their medical careers, have had minimal exposure to the varied medical speciality choices available. Considering this, it is uncertain how predictive of future roles their preferences at this time would be. Despite this the differences in empathy scoring according to future speciality preference at this stage, seems to be a consistent finding [37].

This study employed a scalar tool, JSE, the most commonly used for measuring empathy [41]. Other non-scalar methods, such as patients’ description of physicians’ empathy, may be used in parallel. One limitation of the JSE tool is its reliance on self-reporting of behavior, which may be weakly correlated with actual action and behavior [42]. Students’ responses may have inherent bias to create a favorable impression. It is encouraging to observe increased empathy scores overall, however, the question remains as to whether this statistically significant difference in empathy scores translates into real-life significance in students’ empathy. Further, qualitative studies are needed to evaluate this, particularly considering the perspective of patients, the recipients of student empathy and compassion.

Three participants’ responses were removed from analysis due to very low and discrepant scores, consistent with having misread negative statements and answering them in reverse. To avoid bias, these participants were not asked to repeat the evaluation or alter their responses. The potential for other students having misunderstood statements cannot be excluded. The survey was conducted in English, the language of medical instruction in Nepal, however, as most participants are non-native English speakers the potential for miscomprehension remains, particularly with negatively worded statements. Future use of the Jefferson Scale must consider this phenomenon and its translation into the Nepali language may be important for future use.

Our study demonstrates the positive impact of medical humanities on student empathy scores. Considering the background of low empathy scores amongst Nepali and South Asian medical students [9–12], these findings are encouraging. However, it remains to be seen whether, and how long this change persists, against the trend of declining empathy as training progresses. It is unlikely that a single Medical Humanities module is able to fully counteract such decline. Future cohort analysis will address this question.

## Conclusions

In conclusion, medical humanities teaching is in its infancy in Nepal and South Asia. This study demonstrates the positive impact of a medical humanities module on student empathy in this context and may be a start in addressing the problem of low empathy among health care workers. Medical educators in Nepal and South Asia should consider incorporating Medical Humanities within their curricula.

## Supplementary Information

**Additional file 1.** Dataset -A Quantitative Evaluation of Empathy Using JSE-S Tool, Before and After a Medical Humanities Module, Amongst First-year Medical Students in Nepal 

**Additional file 2.** Medical Humanities Module - Selected Contents 

## Data Availability

The dataset used and analyzed during the current study is available at the supplementary material section as a supplementary data file. The authors may be contacted for help with accessing the dataset.

## Abbreviations

IRI: Interpersonal Reactivity Index
JSE-S: Jefferson Scale of Empathy- Student Version
MBBS: Bachelor of Medicine and Bachelor of Surgery
PAHS: Patan Academy of Health Sciences
SD: standard deviation

## Declarations

We confirm that all methods were carried out in accordance with relevant guidelines and regulations.

All participants gave fully informed written consent to participate at the start of the study.

MS, RG were involved in designing the curriculum and the Medical Humanities course at PAHS. MS, RG, AHD, AA teach ethics and medical humanities at PAHS.
