# Correction to: A quantitative evaluation of empathy using JSE-S Tool, before and after a Medical Humanities Module, amongst first-year medical students in Nepal

**DOI:** 10.1186/s12909-022-03266-1

**Published:** 2022-03-29

**Authors:** Krishna Bahadur G. C., Amit Arjyal, Amanda Helen Douglas, Madhusudan Subedi, Rajesh Gongal

**Affiliations:** 1 Department of Community Health Sciences, Patan Academy of Health Sciences, GPO Box 26500 Ward No.5, Lalitpur Metropolitan City, Bagmati Province Nepal; 2 Department of General Practice and Emergency Medicine, Patan Academy of Health Sciences, Lalitpur, Nepal; 3 Department of Surgery, School of Medicine, Patan Academy of Health Sciences, Lalitpur, Nepal

**Correction to: BMC Med Educ 22, 159 (2022)**

**https://doi.org/10.1186/s12909-022-03188-y**

Following publication of the original article [1], we have been informed that first name and last name has been swapped for author Krishna Bahadur G. C.

The author group has been updated above and the original article has been corrected.
