# Supplementary material for: A quantitative evaluation of empathy using JSE-S Tool, before and after a Medical Humanities Module, amongst first-year medical students in Nepal
Source: BMC Med Educ. 2022 Mar 8;22:159. doi: 10.1186/s12909-022-03188-y (PMC8903097; doi:10.1186/s12909-022-03188-y)
Supplement: Supplementary file 2 — Additional file 2. Medical Humanities Module - Selected Contents [file 12909_2022_3188_MOESM2_ESM.pdf]

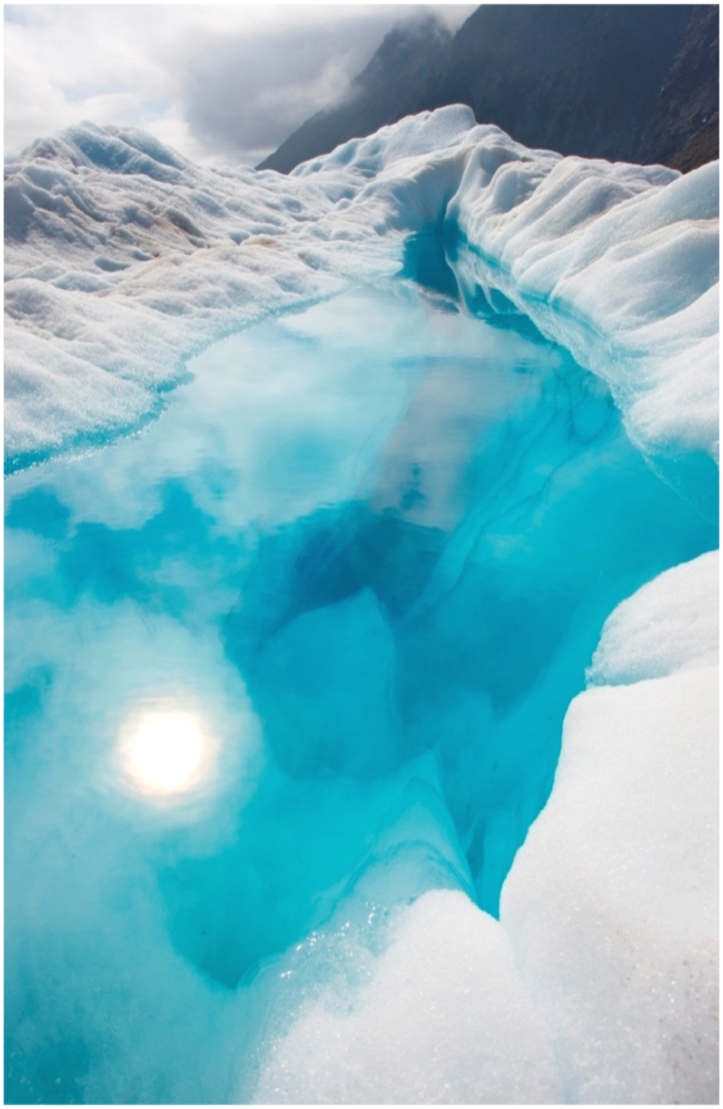

Medical Humanities

FOUNDATION BLOCK

MBBS Program

Patan Academy of Health Sciences

2019

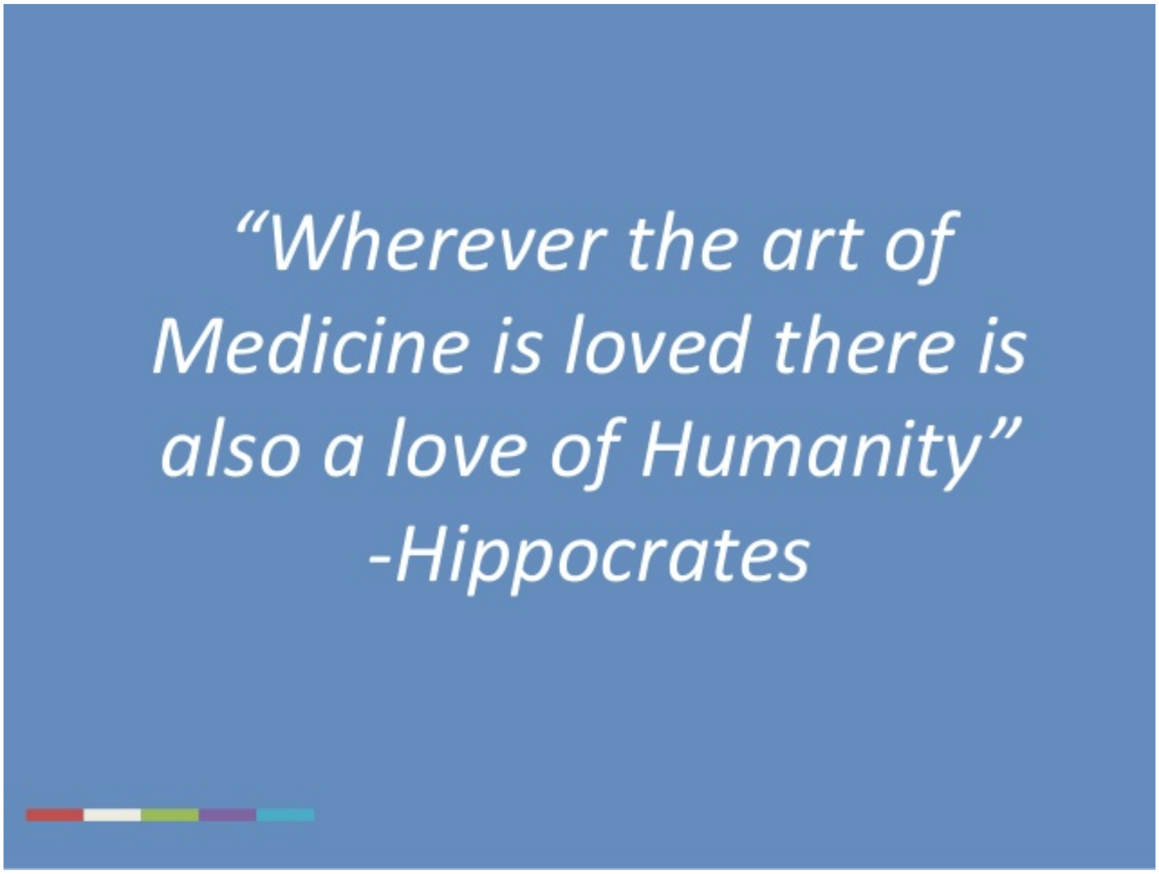

Detailed description of the Medical Humanities Course at PAHS

The course employs various media such as: art, photography, literature, film and poetry. Stimulus material is provided and students engaged in active learning through small group discussion, presentations, poster design and drama. Students undertake a disability exposure: inhabiting the roles of carers and physically disabled people, they visit the local area. After the activity students reflect on their experience. At the end of the course students produce individual written reflections and group dramas, exploring their experiences and learning. Here are some sessions which involve readings from essays, books and poetry:

Session I

Letter from a dying girl from Jumla to Minister for Health, Nepal

स्वास्थ्य मन्त्री थापालाई मृत्यु कुरिरहेकी १९ वर्षे युवतीको पत्र

स्वास्थ्य खबरपत्रिका

२०७३ असोज ८ शनिबार११:४६:५४ प्रकाशित

सन्ध्या शाही जुम्ला

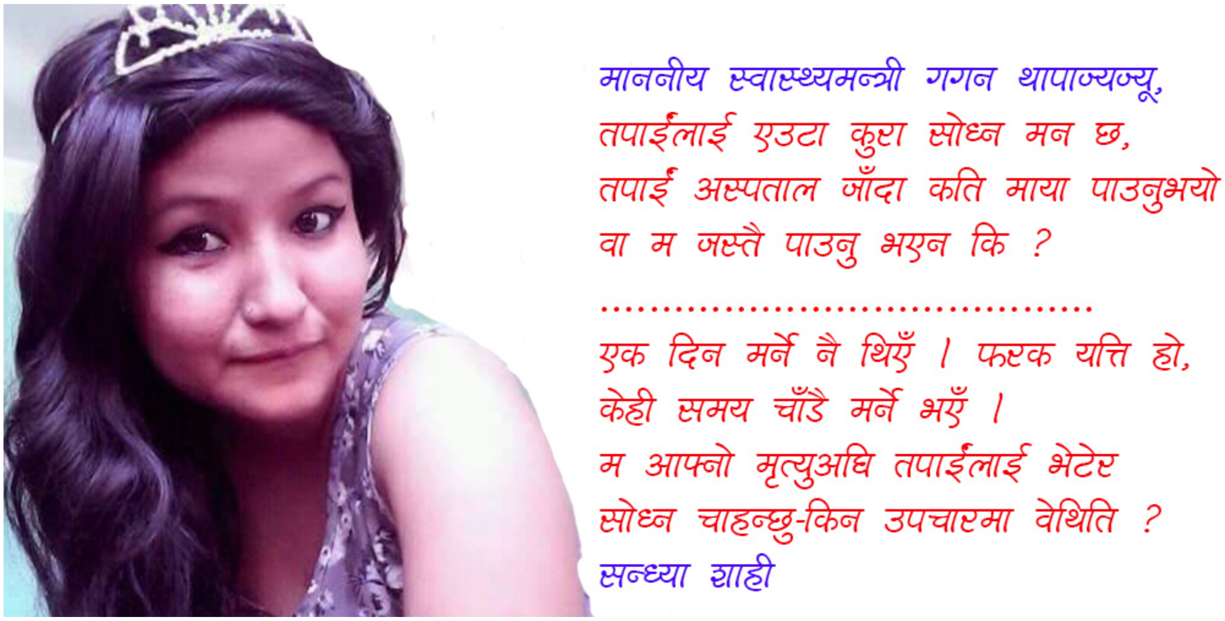

सन्ध्याशाही, जुम्ला

माननीय स्वास्थ्य मन्त्री, गगन थापाज्यू, नमस्कार।

जुनदिन मलाई भक्तपुर क्यान्सर हस्पिटलले मृत्युको पुर्जा दिएर घरमा बसेर मृत्यु कुर्न भन्यो।त्योदिन, मेरो यो उन्नाईस बर्षे कलिलो मनमा कत्रो पहिरो गयो होला ? त्यो कसैले अनुमान गर्न सक्दैन।एकछिन अघि सम्म देखिएको उज्यालो संसार एकाएक चुक घोप्ट्याएको अँध्यारोमा परिणत भयो। त्यस दिन निकै रोएँ।म सँग रुनु बाहेकको विकल्पनै थिएन।शब्दमा व्यक्त गर्न सक्दिनँ त्यो पीडा।रुनु, रुनु र रुनु बाहेक म सँग न त्यो दिन कुनै उपाय थियो न अहिले छ।

मलाई हरेक मानिसलाई जस्तै बाँच्ने, खेल्ने, रमाउने, घुमन्ने अरू के के गर्ने रहर थिए।अनगिन्ती सपनाहरु थिए।जब क्यान्सर फोर्थ स्टेजमा पुगेको थाहा भयो।त्यही दिन देखि जीवनमा रात परेको छ तर सपना देख्न नमिल्ने र नसकिने रात।

निदाउँदा पनि देखिने सपना एउटै हो मृत्युको ।भनिएको छ, म अबको पुगनपुग तीन महिना बाच्न सक्छु अ रे। यो शब्द यस्तो तीर भएको छ कि जुन हरपल मेरो मुटु घोच्न आइपुग्छ।

माननीय मन्त्रीज्यू जीवनको आशा मारिसकेकी एउटी केटीले किन तपाईंलाई पत्र लेखीरहेकी छु भने तपाईंबाट केही आशा छ।यो देशको सिस्टम, यो देशको भुगोल र अन्य कारणले कसरी यो अवस्थामा पुगें भन्ने बताउँदैछु।जसले तपाईंलाई नीति बनाउन सहयोग पुग्ला।जसकाकारण आगामी दिनमा म जस्तै कलिला युवतीहरुले १९ वर्षमै जीवनको आशा मार्नु पर्ने दिनको अन्त्य हुनसक्छ।यो गराउने ठाउँमा तपाईं हुनुहुन्छ।

म अहिले विछ्यौनबाट त्यो दिन अर्था त्मेरो मरणको दिन कल्पिरहेकी े छु।साँच्चि त्यस दिन मेरा सारा आफन्त कसरी रोलान्।कति भक्कानिएलान्।म कल्पनै गर्न सक्दिनँ।मुख्य

कुरा आफूले खाईनखाई यो उन्नाईस बर्ष सम्म हुर्काउने मेरो बाआमाको मनमा कत्रो पहिरो जाला ?

मलाई हरेक मानिसलाई जस्तै बाँच्ने, खेल्ने, रमाउने, घुमन्ने अरू केके गर्ने रहर थिए। अनगिन्ती सपनाहरु थिए। जब क्यान्सर फोर्थस्टेजमा पुगेको थाहा भयो, त्यही दिन देखि जीवनमा रात परेको छ तर सपना देख्न नमिल्ने र नसकिने रात।

छोरी ल्याब टेक्निसिएन बनेर धेरै भन्दा धेरैको रोग पहिचानमा सघाउली भन्ने आशा थियो। तर, म आफ्नै रोग ढिलो पहिचान भएका कारण जीवनसँग थहार्दैगइरहेकी छु।

उनीहरुको यो आशामा कत्रो बाढी पहिरो उर्लिएला त्यो दिन। उनिहरूलाई सम्झाउने मानिसहरूले के भनेर सम्झाउलान् ? सम्झिँदा पनि अत्यास लागिरहेको छ।

म नेपालको अति दुर्गम भुगोल कर्णालीमा जन्मिएँर हुर्कें।जहाँ अन्गिनती दुखहरू छन्। अभावहरू छन्। जुन दुःख र अभावले म जस्तालाई थप दुःख सिर्जना गरिदिन्छ । दुःखहरु सँग जित्न खोज्दा खोज्दै हारिदिन्छौं जिन्दगीको लडाइँ।मैले जस्तै गरी धेरैले पर्खिनु पर्छ मृत्यु।

बाँच्ने संभावना हुँदाहुँदै मृत्यु कुर्ने कर्णालीवासीको तथ्यांक निकाल्ने कसैले प्रयास गरेको छ ? यदि प्रयास गरेको थियो भने देखिन्थ्यो भयाबह चित्र।उपचार नपाएर मर्नु त अहिले पनि नियमित नियती हो त्यहाँका बासिन्दाको।त्यहाँ रोग पत्ता लगाउने उपाय छैन, शहरमा आएर उपचार गराउने सक्ने कर्णाली बासीको हैसियत छैन।

जो दुई छाक खान त्यत्रा दुःखका पहाड फोडिरहेका हुन्छ न्तिनीहरुले महंगो उपचार कसरी कल्पना गर्न सक्छन्।त्यस पछि निर्विकल्प मृत्यु रोज्नु पर्छ।

निको नहुने अवस्थामा पत्ता लाग्यो मेरो रोग।तर पनि उपचारको आशा गर्यो परिवारले।बेच्यो ओत लाग्ने घर पनि।अहिले जुम्लाबाट चाबहिल आएर मेरैलागि डेरामा बसिरहेका छौं सपरिवार।खाउँ–खाउँ, लाउँ–लाउँ भन्ने उमेरकी सन्तानको मृत्यु कुर्नु जस्तो सकस यो दुनियाँमा अरु के हुन सक्ला मन्त्रीज्यू।तर, मेरो परिवार त्यही गरिरहेको छ अहिले जुन मलाई सम्झिँदा पनि अत्यास लाग्छ।

कर्णाली जस्ता दुर्गम ठाउँमा जन्मिनुको दुःख कसैले केहीन केही पाइरहेको छ।जस्तो कि म जब क्यान्सर रोगी भएँ।

मैले देखेका बेथितिहरूले गर्दा देशको माया छ र यो सिस्टम प्रति घृणा छ। हो म मृत्यु अघि उपचारका क्रममा पाएका दुख हजुरलाई मर्नु अघि सुनाउन चाहन्छु। किनभने म जस्ता नागरिकले उपचारमा रहेका वेथितिका कारण कलिलैमा ज्यान गुमाउन नपरोस्।

हातमा पलाएको खटिराको उपचार गर्न म शिक्षण अस्पताल महराजगञ्ज पुगेकी थिएँ, केही समय पहिले।कर्णाली मै ती खटिरा के हुन्न किन चाँडो उपचार गराउनु पर्छ भन्ने सम्मका अस्पताल हुँदाहुन्त मेरो बेलैमा रोग पहिचान हुन्थ्यो र उपचार हुन्थ्यो।तर, धेरै थोक मिलाएर काठमाडौं आइपुग्न समय लाग्यो। जुनदिन ती त क्यान्सरका लक्षण हुन्भन्ने थाहा भयो, त्यो दिन निकै ढिलोभइसकेको थियो।शिक्षण अस्पतालले हातका औंला काटे पछि क्यान्सर ठिक हुन सक्ने बतायो।

किनभने हातको क्यान्सर दोस्रो स्टेजमा रहेछ।काटियो। तर, पेटमा पनि क्यान्सर भइसकेको भने निकै ढिलोगरी थाहा भयो, अन्ति मस्टेजमा पुगेपछि।यहाँ नेर कहीँनकहीँ उपचार प्रणालीको दोषछ किभन्ने लाग्छ मलाई।त्यो तपाईं आफैं बुझ्नु होला।

अहिले म अनुभव गरिरहेकी छु, दुर्गम हर नागरिकका लागि कति पिडादायिक भुगोल रहेछ ।जब मैले आफू बाँच्न हरेक व्यक्तिसँग आलापविलाप गरें, दुर्गम त मान्छेको सत्रु नै रहेछ भनेर बुझें। मैले अनुभव गरेका कुरामा धेरै सत्यता छ।त्यसैले तपाईंले मेरो मृत्युबारे जानकार गराउन लागिरहेको छु।अनि यो पत्र सकिनसकि लेखिरहेकी छु।

मैले पाउनु पर्ने केमो लिइरहेकी छु।त्यसैले आफ्ना लागि मैले तपाईंसँग केही माग्नु छैन।जीवन नै त्याग्ने बखतमा पुगेकी म अरु के माग्न सक्छु र ?

तपाईंलाई यसकारण पत्र लेखिरहेकी छु कि मैले सानै देखि तपाईको बारे सुनेको छु।तपाईबारे पढेको छु।तपाईं जस्ता अरू पनि धेरै मानिसहरू हुँदाहुन्त यो देशमा देश अर्कै हुन्थ्यो जस्तो लाग्छ।तपाईंले मलाई चिन्नु हुन्त तर मलाई भने तपाईं नजिकको जस्तो लाग्छ।त्यसैले चिठी लेखें, आफ्नै मृत्युको जानकारी दिन।जसले तपाईंलाई म जस्ता तपाईका देश भरि रहेका आफन्त जोगाउन सहयोग पुगोस्।

मैले पाउनु पर्ने केमो लिइरहेकी छु।त्यसैले आफ्ना लागि मैले तपाईं सँग केही माग्नु छैन।जीवन नै त्याग्ने बखतमा पुगेकी म अरु के माग्न सक्छु र ?

माननीय मन्त्रीज्यू , म पनि यहि देशमा जन्मे।यही देशमा हुर्कें।जहाँ तपाईं पनि जन्मनु भो र हुर्कनु भो।तर तपाईंलाई एउटा कुरा सोध्न मन छ।तपाईंलाई एउटा कुरा सोध्न मन छ , तपाईं बिरामी हुँदा वा कोही बिरामी लिएर अस्पताल जाँदा कति माया पाउनुभयो ? सम्मान पाउनुभयो ? वा म जस्तै पाउनु भएन कि ? मैले त कति पनि पाइनँ अस्पतालमा माया।कहीँ अनुभव गरिनँ, बिरामीलाई गरिने न्युनतम सम्मानको।

मैले उपचारका क्रममा यति दुःख पाएँ कि अनुभव भयो म त यो देशको नागरिक नै होइन जस्तो।मेरो आँखाले कति हो कति बेथितिहरू देखेकी छु अस्पतालमा जुन बताउने हालतमा छैन म।सायद तपाईंलाई थाहा पनि होला त्यसैले भन्नु पर्दन।त्यो बेथितिको सिकार म पनि भएँ।र, सम्झे बेथितिहरूले म जस्ता अरू पनि दिनदिनै मरिरहेका होलान्।यी कुरा जानकारी गराउन मन लाग्यो र चिठी लेखिरहेको छु।

हुन त म एक दिन मर्ने नै थिएँ।फरक यत्ति हो, केही समय चाँडै मर्ने भएँ।मृत्यु कुरिरहेकी युवती सँग कति दुःख छन्होला त्यो हजुरले महशुस गर्न सक्नु हुन्छ।

म आफ्नो मृत्यु अघि तपाईंलाई भेटेर किन यस्तो भइरहेको छ भनेर सोध्न चाहन्छु तर भेट कति संभव वा असंभव मैले बुझेकी छैन।

भेटेर एउटै कुरा सोध्न मन छ, उपचार यस्तो लापरबाही किन हुन्छ हाम्रो देशमा ? यत्ति बुझेर मर्न चाहान्छु।

माननीय मन्त्रीज्यू, म बाँचेर पनि देशको लागि के गर्नसक्थे होला र।तर म मर्ने पक्का भएपछि देशको हो वा आफ्नो खुब माया लागिरहेको छ।

मैले देखेका बेथितिहरूले गर्दा देशको माया छ र यो सिस्टम प्रतिघृणा छ।हो म मृत्यु अघि उपचारका क्रममा पाएका दुख हजुरलाई मर्नु अघि सुनाउन चाहन्छु।किनभने म जस्ता नागरिकले उपचारमा रहेका वेथिति का कारण कलिलैमा ज्यान गुमाउन नपरोस्।

तपाईलाई मेरा धैरै कुराहरू भन्नु छ।जुन कुराहरू म चाहेर पनि यो पत्रमा लेख्न सक्दिनँ।भन्न सक्दिनँ।घाउ पनि दुखेर बढिरहेको छ।पिडाले मुटु काँपिरहेको छ। म यो भन्दा बढि लेख्न सकिनँ।कृपया सक्नुहुन्छ भने म मर्नु पहिले म हजुरसगँ सबै कुरा भन्ने समय पाउँ।सक्नुहुन्न भने यो काम चहि गरिदिनु होला।देशका हर नागरिकलाई मृत्यु दिने बेथितिहरूको अन्त होस्।लापरबाहिले एक बर्ष पछि मर्ने नागरिक एक बर्ष अघि नमरोस्।

Exercise

Ask one of the students, preferably female student, to read the letter aloud in the class.

How do you feel after reading the letter? Tell us your feeling.

Allow 3-4 students to express what they feel.

What are the important issues arising from this letter?

Just bring out points without going into depth (at present)

<u>Issues for discussion</u>

Problem with health system

Problem with health service especially in remote areas

Difficult life in remote areas

Injustice to people in remote area

Feeling of people who are dying

Hope

Love for the country

How do people die?

Are health professional treating dying people well?

Expectation of patients

Divide into small groups and allow them to discuss in one issue.

Presentation in plenary

Conclusion

End of Session I

Session II

A book by a young trainee neurosurgeon at the end of his training who developing incurable lung cancer.

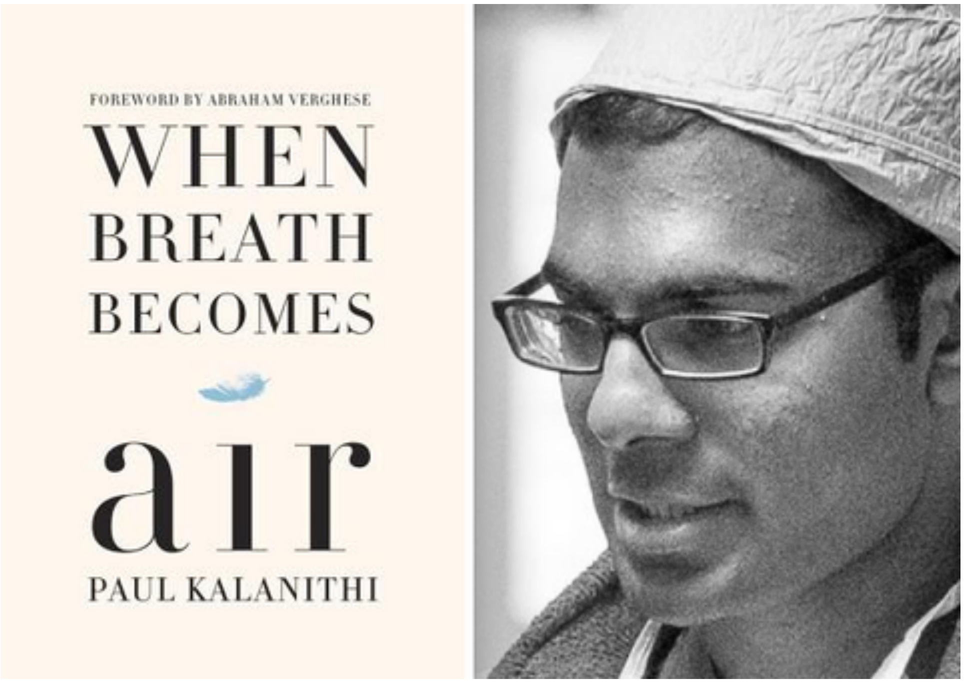

I FLIPPED THROUGH THE CT scan images, the diagnosis obvious: the lungs were matted with innumerable tumors, the spine deformed, a full lobe of the liver obliterated. Cancer, widely disseminated. I was a neurosurgical resident entering my final year of training. Over the last six years, I'd examined scores of such scans, on the off chance that some procedure might benefit the patient. But this scan was different: it was my own.

I wasn't in the radiology suite, wearing my scrubs and white coat. I was dressed in a patient's gown, tethered to an IV pole, using the computer the nurse had left in my hospital room, with my wife, Lucy, an internist, at my side. I went through each sequence again, scrolling from top to bottom, then left to right, then front to back, as if I might find something that would change the diagnosis.

We lay together on the hospital bed.

Lucy, quietly, as if reading from a script: "Do you think there's any possibility that it's something else?"

"No," I said.

We held each other tightly, like young lovers. In the past year we'd both suspected, but refused to believe, or even discuss, that a cancer was growing inside me.

LYING NEXT TO LUCY in the hospital bed, both of us crying, the CT scan images still glowing on the computer screen, that identity as a physician—my identity—no longer mattered. With the cancer having invaded multiple organ systems, the diagnosis was clear. The room was quiet. Lucy told me she loved me. "I don't want to die," I said. I told her to remarry, that I couldn't bear the thought of her being alone. I told her we should refinance the mortgage immediately. We started calling family members. At some point, Victoria came by the room, and we discussed the scan and the likely future treatments. When she brought up the logistics of returning to residency, I stopped her.

"Victoria," I said, "I'm never coming back to this hospital as a doctor. Don't you think?"

One chapter of my life seemed to have ended; perhaps the whole book was closing. I found myself lost and confused. Severe illness wasn't life-altering, it was life-shattering.

My brother Jeevan had arrived at my bedside. "You've accomplished so much," he said. "You know that, don't you?"

I sighed. He meant well, but the words rang hollow. My life had been building potential, potential that would now go unrealized. I had planned to do so much, and I had come so close. I was physically debilitated, my imagined future and my personal identity collapsed, and I faced the same existential quandaries my patients faced. The lung cancer diagnosis was confirmed. My carefully planned and hard-won future no longer existed. Death, so familiar to me in my work, was now paying a personal visit. Here we were, finally face-to-face, and yet nothing about it seemed recognizable. Standing at the crossroads where I should have been able to see and follow the footprints of the countless

patients I had treated over the years, I saw instead only a blank, a harsh, vacant, gleaming white desert, as if a sandstorm had erased all trace of familiarity.

The sun was setting. I would be discharged the next morning. An oncology appointment was set for later in the week, but the nurse told me my oncologist was going to drop by that night, before leaving to pick up her kids. Her name was Emma Hayward, and she wanted to say hello before the initial office visit. My parents and brothers were scattered about the room, not saying much, while Lucy sat by the bed, holding my hand. The door opened and in she walked, her white coat showing the wear of a long day but her smile fresh. Trailing behind her were her fellow and a resident. Emma was only a few years older than I, her hair long and dark, but as is common to all those who spend time with death, streaked with gray. She pulled up a chair.

“Hi, my name is Emma,” she said. “I’m sorry to have to be so brief today, but I wanted to come by and introduce myself.”

“I’m sorry this is happening to you,” she said. “To all of you. There will be a lot of time to talk in a couple days. I went ahead and had the lab start running some tests on your tumor sample, which will help guide therapy. Treatment may be chemotherapy or not, depending on the tests.”

Two days later, Lucy and I met Emma in the clinic. My parents hovered in the waiting room. The medical assistant took my vitals. Emma and her nurse practitioner were remarkably punctual, and Emma pulled up a chair in front of me, to talk face-to-face, eye-to-eye.

“Most of your tests are back,” Emma said. “You have a PI3K mutation, but no one’s sure what that means yet. The test for the most common mutation in patients like you, EGFR, is still pending. I’m betting that’s what you have, and if so, there’s a pill called Tarceva that you can take instead of chemotherapy. That result should be back tomorrow, Friday, but you’re sick enough that I’ve set you up for chemo starting Monday in case the EGFR test is negative.”

“With chemo, our main decision will be carboplatin versus cisplatin. In isolated studies, head-to-head, carboplatin is better tolerated. Cisplatin has potentially better results but much worse toxicity, especially for the nerves, Do you have any thoughts?”

“I’m less worried about protecting my hands for surgery,” I said. “There’s a lot I can do with my life. If I lose my hands, I can find another job, or not work, or something.”

She paused. “Let me ask this: Is surgery important to you? Is it something you want to do?”

“Well, yes, I’ve spent almost a third of my life preparing for it.”

"Okay, then I'm going to suggest we stick with the carboplatin. I don't think it will change survival, and I do think it could dramatically change your quality of life. Do you have any other questions?"

I began to realize that coming in such close contact with my own mortality had changed both nothing and everything. Before my cancer was diagnosed, I knew that someday I would die, but I didn't know when. After the diagnosis, I knew that someday I would die, but I didn't know when. But now I knew it acutely. The problem wasn't really a scientific one. The fact of death is unsettling. Yet there is no other way to live.

Slowly the medical fog was clearing—at least now I had enough information to dive into the literature. While the numbers were fuzzy, having an EGFR mutation seemed to add around a year of life on average, with the potential for long-term survival; not having it suggested an 80 percent chance of death within two years. Clarifying the rest of my life was going to be a process.

The next day, Lucy and I went to the sperm bank, to preserve gametes and options. We had always planned to have kids at the end of my residency, but now…The cancer drugs would have an unknown effect on my sperm, so to keep a chance of having children, we'd have to freeze sperm before I started treatment.

I began to look forward to my meetings with Emma. In her office, I felt like myself, like a self. Outside her office, I no longer knew who I was. Because I wasn't working, I didn't feel like myself, a neurosurgeon, a scientist—a young man, relatively speaking, with a bright future spread before him. Debilitated, at home, I feared I wasn't much of a husband for Lucy. I had passed from the subject to the direct object of every sentence of my life. In fourteenth-century philosophy, the word patient simply meant "the object of an action," and I felt like one. As a doctor, I was an agent, a cause; as a patient, I was merely something to which things happened. But in Emma's office, Lucy and I could joke, trade doctor lingo, talk freely about our hopes and dreams, try to assemble a plan to move forward. Two months in, Emma remained vague about any prognostication, and every statistic I cited she rebuffed with a reminder to focus on my values. Though I felt dissatisfied, at least I felt like somebody, a person, rather than a thing exemplifying the second law of thermodynamics (all order tends toward entropy, decay, etc.).

Flush in the face of mortality, many decisions became compressed, urgent and unreceding. Foremost among them for us: Should Lucy and I have a child? Even if our marriage had been strained toward the end of my residency, we had always remained very much in love. Our relationship was still deep in meaning, a shared and evolving vocabulary about what mattered. If human relationality formed the bedrock of meaning, it seemed to us that rearing children added another dimension to that meaning. It had been something we'd always wanted, and we were both impelled by the instinct to do it still, to add another chair to our family's table.

Both of us yearning to be parents, we each thought of the other. Lucy hoped I had years left, but understanding my prognosis, she felt that the choice—whether to spend my remaining time as a father—should be mine.

“What are you most afraid or sad about?” she asked me one night as we were lying in bed.

“Leaving you,” I told her.

I knew a child would bring joy to the whole family, and I couldn’t bear to picture Lucy husbandless and childless after I died, but I was adamant that the decision ultimately be hers: she would likely have to raise the child on her own, after all, and to care for both of us as my illness progressed.

“Will having a newborn distract from the time we have together?” she asked. “Don’t you think saying goodbye to your child will make your death more painful?”

“Wouldn’t it be great if it did?” I said. Lucy and I both felt that life wasn’t about avoiding suffering.

After so many years of living with death, I’d come to understand that the easiest death wasn’t necessarily the best. We talked it over. Our families gave their blessing. We decided to have a child. We would carry on living, instead of dying.

A few months after starting Tarceva, My tumor was relatively controlled , I was able to go back to what I loved doing most, surgery.

I was operating until late at night or into the early morning, fixated on graduation, my diagnosis nine months in the past. My body was taking a beating. I was too tired to eat when I got home. I had been slowly upping the dose of Tylenol and NSAIDs and antiemetics. I had developed a persistent cough, presumably caused by scarring from the dead tumor in my lungs. I only had to keep up this relentless pace for a couple more months, I told myself, and then I would graduate from residency and settle into the comparatively calmer role of a professor.

I thought about what Emma had told me. I had gone from being unable to believe I could be a surgeon to being one, a transformation that carried the force of religious conversion. She had always kept this part of my identity in mind, even when I couldn’t. She had done what I had challenged myself to do as a doctor years earlier: accepted mortal responsibility for my soul and returned me to a point where I could return to myself. I had attained the heights of the neurosurgical trainee, set to become not only a neurosurgeon but a surgeon-scientist. Every trainee aspires to this goal; almost none make it.

I could hear Emma’s voice again: You have to figure out what’s most important to you.

If I no longer sought to fly on the highest trajectory of neurosurgeon and neuroscientist, what did I want?

To be a father?

To be a neurosurgeon?

To teach?

I didn't know. But if I did not know what I wanted, I had learned something, something not found in Hippocrates: the physician's duty is not to stave off death or return patients to their old lives, but to take into our arms a patient and family whose lives have disintegrated and work until they can stand back up and face, and make sense of, their own existence.

My own hubris as a surgeon stood naked to me now: as much as I focused on my responsibility and power over patients' lives, it was at best a temporary responsibility, a fleeting power. Once an acute crisis has been resolved, the patient awakened, extubated, and then discharged, the patient and family go on living—and things are never quite the same. A physician's words can ease the mind, just as the neurosurgeon's scalpel can ease a disease of the brain. Yet their uncertainties and morbidities, whether emotional or physical, remain to be grappled with.

Emma hadn't given me back my old identity. She'd protected my ability to forge a new one. And, finally, I knew I would have to.

……

I hopped out of the CT scanner, seven months since I had returned to surgery. This would be my last scan before finishing residency, before becoming a father, before my future became real.

"Wanna take a look, Doc?" the tech said.

"Not right now," I said. "I've got a lot of work to do today."

It was already six P.M. I had to go see patients, organize tomorrow's OR schedule, review films, dictate my clinic notes, check on my post-ops, and so on. Around eight P.M., I sat down in the neurosurgery office, next to a radiology viewing station. I turned it on, looked at my patients' scans for the next day—two simple spine cases—and, finally, typed in my own name. I zipped through the images as if they were a kid's flip-book, comparing the new scan to the last. Everything looked the same, the old tumors remained exactly the same…except, wait.

I rolled back the images. Looked again.

There it was. A new tumor, large, filling my right middle lobe. It looked, oddly, like a full moon having almost cleared the horizon. Going back to the old images, I could make out the faintest trace of it, a ghostly harbinger now brought fully into the world.

I was neither angry nor scared. It simply was. It was a fact about the world, like the distance from the sun to the earth. I drove home and told Lucy. It was a Thursday night,

and we wouldn't see Emma again until Monday, but Lucy and I sat down in the living room, with our laptops, and mapped out the next steps: biopsies, tests, chemotherapy. The treatments this time around would be tougher to endure, the possibility of a long life more remote. Neurosurgery would be impossible for a couple of weeks, perhaps months, perhaps forever. But we decided that all of that could wait to be real until Monday. Today was Thursday, and I'd already made tomorrow's OR assignments; I planned on having one last day as a resident.

xxxxxxxxxxxxxxxxxxxxxxxxxxxxxxxxxxxxxxxxxxxxxxxxxxxxxxxxxxxxxxxxxxxxxxxxx

On the way home from the appointment with Emma, Lucy's mom called to say they were headed to the hospital. Lucy was in labor. ("Make sure you ask about the epidural early," I told her. She had suffered enough.) I returned to the hospital, pushed by my father in a wheelchair. I lay down on a cot in the delivery room, heat packs and blankets keeping my skeletal body from shivering. For the next two hours, I watched Lucy and the nurse go through the ritual of labor. As a contraction built up, the nurse counted off the pushing: "And a one two three four five six seven eight nine and a ten!"

Lucy turned to me, smiling. "It feels like I'm playing a sport!" she said.

I lay on the cot and smiled back, watching her belly rise. There would be so many absences in Lucy's and my daughter's life—if this was as present as I could be, then so be it.

Sometime after midnight, the nurse nudged me awake. "It's almost time," she whispered. She gathered the blankets and helped me to a chair, next to Lucy. The obstetrician was already in the room, no older than I. She looked up at me as the baby was crowning. "I can tell you one thing: your daughter has hair exactly like yours," she said. "And a lot of it." I nodded, holding Lucy's hand during the last moments of her labor. And then, with one final push, on July 4, at 2:11 A.M., there she was. Elizabeth Acadia—Cady; we had picked the name months before.

"Can we put her on your skin, Papa?" the nurse asked me.

"No, I'm too c-c-cold," I said, my teeth chattering. "But I would love to hold her."

They wrapped her in blankets and handed her to me. Feeling her weight in one arm, and gripping Lucy's hand with the other, the possibilities of life emanated before us. The cancer cells in my body would still be dying, or they'd start growing again.

Yet there is dynamism in our house.

Day to day, week to week, Cady blossoms: a first grasp, a first smile, a first laugh. A brightening newness surrounds her. As she sits in my lap smiling, enthralled by my tuneless singing, an incandescence lights the room.

Time for me is now double-edged: every day brings me further from the low of my last relapse but closer to the next recurrence—and, eventually, death. Perhaps later than I think, but certainly sooner than I desire. There are, I imagine, two responses to that realization. The most obvious might be an impulse to frantic activity: to "live life to its fullest," to travel, to dine, to achieve a host of neglected ambitions. Part of the cruelty of cancer, though, is not only that it limits your time; it also limits your energy, vastly reducing the amount you can squeeze into a day. It is a tired hare who now races. And even if I had the energy, I prefer a more tortoise like approach. I plod, I ponder. Some days, I simply persist.

Everyone succumbs to finitude. I suspect I am not the only one who reaches this state. Most ambitions are either achieved or abandoned; either way, they belong to the past. The future, instead of the ladder toward the goals of life, flattens out into a perpetual present. Money, status, all the vanities described hold so little interest: a chasing after wind, indeed.

Yet one thing cannot be robbed of her futurity: our daughter, Cady. I hope I'll live long enough that she has some memory of me. Words have a longevity I do not. I had thought I could leave her a series of letters—but what would they say? I don't know what this girl will be like when she is fifteen; I don't even know if she'll take to the nickname we've given her. There is perhaps only one thing to say to this infant, who is all future, overlapping briefly with me, whose life, barring the improbable, is all but past.

That message is simple:

When you come to one of the many moments in life where you must give an account of yourself, provide a ledger of what you have been, and done, and meant to the world, do not, I pray, discount that you filled a dying man's days with a sated joy, a joy unknown to me in all my prior years, a joy that does not hunger for more and more but rests, satisfied. In this time, right now, that is an enormous thing.

Epilogue

PAUL DIED ON MONDAY, March 9, 2015, surrounded by his family, in a hospital bed roughly two hundred yards from the labor and delivery ward where our daughter, Cady, had entered the world eight months before. Between Cady's birth and Paul's death, if you'd seen us sucking on ribs at our local barbecue restaurant and smiling over a shared beer, a dark-haired baby with long eyelashes napping in her stroller beside us, you'd never have guessed that Paul likely had less than a year to live, nor that we understood that.

It was around Cady's first Christmas, when she was five months old, that Paul's cancer began to resist the third-line drugs recommended after Tarceva and then chemotherapy had stopped working. His strength waned over the following months, but we continued to experience joyful moments, even in the midst of our sorrow. We hosted cozy dinner parties, held each other at night, and delighted in our daughter's bright eyes and calm

nature. And, of course, Paul wrote, reclining in his armchair, wrapped in a warm fleece blanket. In his final months, he was singularly focused on finishing this book.

By late February, he needed supplemental oxygen to keep his breathing comfortable. I was adding his untouched lunch to the trash can atop his untouched breakfast, and a few hours later I'd add an untouched dinner to the pile. He used to love my breakfast sandwiches—egg, sausage, and cheese on a roll—but with his waning appetite we'd changed to eggs and toast, then just eggs, until even those became intolerable. Even his favorite smoothies, the glasses I filled with a steady stream of calories, were unappetizing.

Bedtime crept earlier, Paul's voice slurred intermittently, and his nausea became unremitting. A CT scan and brain MRI confirmed worsening cancer in Paul's lungs and new tumors that had landed in his brain, including leptomeningeal carcinomatosis, a rare and lethal infiltration that brought with it a prognosis of only several months and the looming shadow of swift neurologic decline. The news hit Paul hard. He said little, but as a neurosurgeon, he knew what lay ahead. Although Paul accepted his limited life expectancy, neurologic decline was a new devastation, the prospect of losing meaning and agency agonizing. We strategized with Paul's oncologist about his top priority: preserving mental acuity as long as possible. We arranged entry into a clinical trial, consultation with a neuro-oncology specialist, and a visit with his palliative-care team to discuss hospice options, all in service of maximizing the quality of his remaining time. My heart swelled even as I steeled myself, anticipating his suffering, worrying that he had only weeks left—if that. I envisioned his funeral as we held hands. I didn't know that Paul would die within days.

We spent Paul's last Saturday with family in the nest of our living room, Paul holding Cady in his armchair; his father on my nursing glider; his mother and I on sofas nearby. Paul sang to Cady and bounced her gently in his lap. She grinned widely, oblivious to the tubing that delivered oxygen to his nose. He didn't write anything that day. The manuscript for this book was only partially finished, and Paul now knew that he was unlikely to complete it—unlikely to have the stamina, the clarity, the time.

The next day, Sunday, we hoped for a continuation of the calm weekend. If Paul felt well enough, we would attend church, then take Cady and her cousin to the baby swings at the park up the hill. We'd continue to absorb the recent painful news, share the sorrow, savor our time together.

But instead, time sped up.

Early Sunday morning, I stroked Paul's forehead and found it scorching with fever, 104 degrees, though he was relatively comfortable and free of other new symptoms. We made it in and out of the emergency room within a few hours, Paul's father and Suman with us, returning home to the rest of the family after starting antibiotics in case of pneumonia (Paul's chest X-ray was dense with tumors, which could obscure an infection). But was this, instead, the cancer progressing rapidly? Paul napped comfortably in the afternoon,

but he was gravely ill. I started to cry as I watched him sleep, then crept out to our living room, where his father's tears joined mine. I already missed him.

Sunday evening, Paul's condition worsened abruptly. He sat on the edge of our bed, struggling to breathe—a startling change. I called an ambulance. When we reentered the emergency room, Paul on a gurney this time, his parents close behind us, he turned toward me and whispered, "This might be how it ends."

"I'm here with you," I said.

The hospital staff greeted Paul warmly, as always. But they moved quickly once they saw his condition. After initial testing, they placed a mask over his nose and mouth to help his breathing via BiPAP, a breathing support system that supplied a strong mechanized flow of air each time he inhaled, doing much of the work of breathing for him.

. He observed. He understood, as a physician, the ominous test results. I understood them, too, walking behind him as he was wheeled to an intensive-care room, one where so many of his own patients had struggled before or after neurosurgery, their families assembled in vinyl chairs by their bedsides. "Will I need to be intubated?" he asked me between BiPAP breaths when we arrived. "Should I be intubated?"

Through the night, Paul discussed that question in a series of conversations with his physicians, his family, and then just me. Around midnight, the critical-care attending, a longtime mentor to Paul, came in to discuss treatment options with the family. BiPAP was a temporary solution, he said. The only remaining intervention would be for Paul to be intubated—put on a ventilator. Was that what he wanted?

The key question quickly came into view: Could the sudden respiratory failure be reversed?

Of concern was whether Paul would remain too ill to ever come off the ventilator—would he be lost to delirium and then organ failure, first mind and then body slipping away? We'd witnessed this agonizing scenario as physicians. Paul explored the alternative: in lieu of intubation, he could choose "comfort care," though death would come more surely and swiftly. "Even if I make it through this," he said, thinking of the cancer in his brain, "I'm not sure I see a future that includes meaningful time." His mother chimed in, desperately. "No decisions tonight, Pubby," she said. "Let's all get some rest." After ensuring his "do not resuscitate" status, Paul agreed. Sympathetic nurses brought him extra blankets. I switched off the fluorescent lights.

Paul managed to doze until sunrise, his father sitting vigil while I napped briefly in an adjacent room, hoping to preserve my mental strength, knowing that the following day might be the hardest of my life. I crept back to Paul's room at six A.M., the lights still low, the intensive-care monitors chiming intermittently. Paul opened his eyes. We talked again about "comfort care"—avoiding aggressive attempts to forestall his decline—and he wondered aloud whether he could go home. He was so ill that I worried he might suffer and die on the way. However, I said I would do everything possible to take him

home if that was most important to him, nodding that yes, comfort care might be the direction we were headed. Or was there some way to re-create home here? Between BiPAP puffs, he answered: “Cady.”

Cady arrived in short order—our friend Victoria had retrieved her from home—and began her own unwitting, cheerful vigil, happily nestled in the crook of Paul’s right arm, tugging at her tiny socks, batting at his hospital blankets, smiling and cooing, unbothered by the BiPAP machine as it continued to blow, keeping Paul alive.

The medical team came by on rounds, discussing Paul’s case outside the room, where his family and I joined them. Paul’s acute respiratory failure was likely rapid cancer progressing. His carbon dioxide level was rising still—a hardening indication for intubation. The family was torn: Paul’s oncologist had phoned in, hopeful that the acute problem could be ameliorated, but the physicians present were less optimistic. I entreated them to weigh in with as much conviction as possible on the chance of reversing his abrupt decline.

“He doesn’t want a Hail Mary,” I said. “If he doesn’t have a chance of meaningful time, he wants to take the mask off and hold Cady.”

I returned to Paul’s bedside. He looked at me, his dark eyes alert above the nose bridge of the BiPAP mask, and said clearly, his voice soft but unwavering, “I’m ready.”

Ready, he meant, to remove the breathing support, to start morphine, to die.

The family gathered together. During the precious minutes after Paul’s decision, we all expressed our love and respect. Tears glistened in Paul’s eyes. He expressed gratitude to his parents. He asked us to ensure that his manuscript be published in some form. He told me a last time that he loved me. The attending physician stepped in with strengthening words: “Paul, after you die, your family will fall apart, but they’ll pull it back together because of the example of bravery you set.” Jeevan’s eyes were trained on Paul as Suman said, “Go in peace, my brother.” With my heart breaking, I climbed into the last bed we would share.

I thought of other beds we’d shared. Eight years prior, as medical students, we’d slept similarly ensconced in a twin bed next to my grandfather as he lay dying at home, having cut our honeymoon short to help with caregiving duties. We awakened every few hours to give him medications, my love for Paul deepening as I watched him lean in and listen closely to my grandfather’s whispered requests. We’d never have imagined this scene, Paul’s own deathbed, so near in our future. Twenty-two months ago, we’d cried in a bed on another floor of this same hospital as we learned of Paul’s cancer diagnosis. Eight months ago, we’d been together here in my hospital bed the day after Cady was born, both napping, the first good, long sleep I’d had since her birth, wrapped in each other’s arms. I thought of our cozy bed empty at home, remembered falling in love in New Haven twelve years earlier, surprised right away by how well our bodies and limbs fit together, and thought of how ever since, we’d both slept best when entwined. I hoped with all I had that he felt that same restful comfort now.

An hour later, the mask and monitors were off, and morphine was flowing through Paul's IV. He was breathing steadily but shallowly, and he appeared comfortable. Nonetheless, I asked him whether he needed more morphine, and he nodded yes, his eyes closed. His mother sat close; his father's hand rested atop his head. Finally, he slipped into unconsciousness.

For more than nine hours, Paul's family—his parents, brothers, sister-in-law, daughter, and I—sat vigil as Paul, unconscious, now drew increasingly halting, infrequent breaths, his eyelids closed, his face unburdened. His long fingers rested softly in mine. Paul's parents cradled Cady and then put her in the bed again to snuggle, nurse, nap. The room, saturated with love, mirrored the many holidays and weekends we had all spent together over the years. I stroked Paul's hair, whispering, "You're a brave Paladin"—my nickname for him—and singing quietly into his ear a favorite jingle we'd made up over the previous months, its core message being "Thank you for loving me." A close cousin and uncle arrived, and then our pastor. The family shared loving anecdotes and inside jokes; then we all took turns weeping, studying Paul's face and each other's with concern, steeped in the preciousness and pain of this time, our last hours all together.

Warm rays of evening light began to slant through the northwest-facing window of the room as Paul's breaths grew more quiet. Cady rubbed her eyes with chubby fists as her bedtime approached, and a family friend arrived to take her home. I held her cheek to Paul's, tufts of their matching dark hair similarly askew, his face serene, hers quizzical but calm, his beloved baby never suspecting that this moment was a farewell. Softly I sang Cady's bedtime song, to her, to both of them, and then released her.

As the room darkened into night, a low wall lamp glowing warmly, Paul's breaths became faltering and irregular. His body continued to appear restful, his limbs relaxed. Just before nine o'clock, his lips apart and eyes closed, Paul inhaled and then released one last, deep, final breath.

Exercise

Introduce the author and the circumstances of his life

A short video

Ask one of the boys to read the part written by Paul.

Ask one of the girls to read the epilogue written by Paul's wife Lucy

Issues: Death and dying. How do we view life? How do we view death? What is good death? Is it important? Medicalization of death.

After completion of reading, ask how students felt about it. Get reaction from three to four students.

What are the important issues arising from this book?

Only points without in-depth discussion.

Organize the points in five or six domains.

Divide the students in several groups and ask them to discuss a point each.

Bring to plenary and elaborate the discussion arising from the plenary.

Session III

Exercise

Continue from the Session II.

Further discussion on death and dying, what is good death?

Conclusion.

Session IV

कविता

बग्दै बग्दै जाने छोरासँग

विवश पोखरेल

भाद्र१७, २०७४

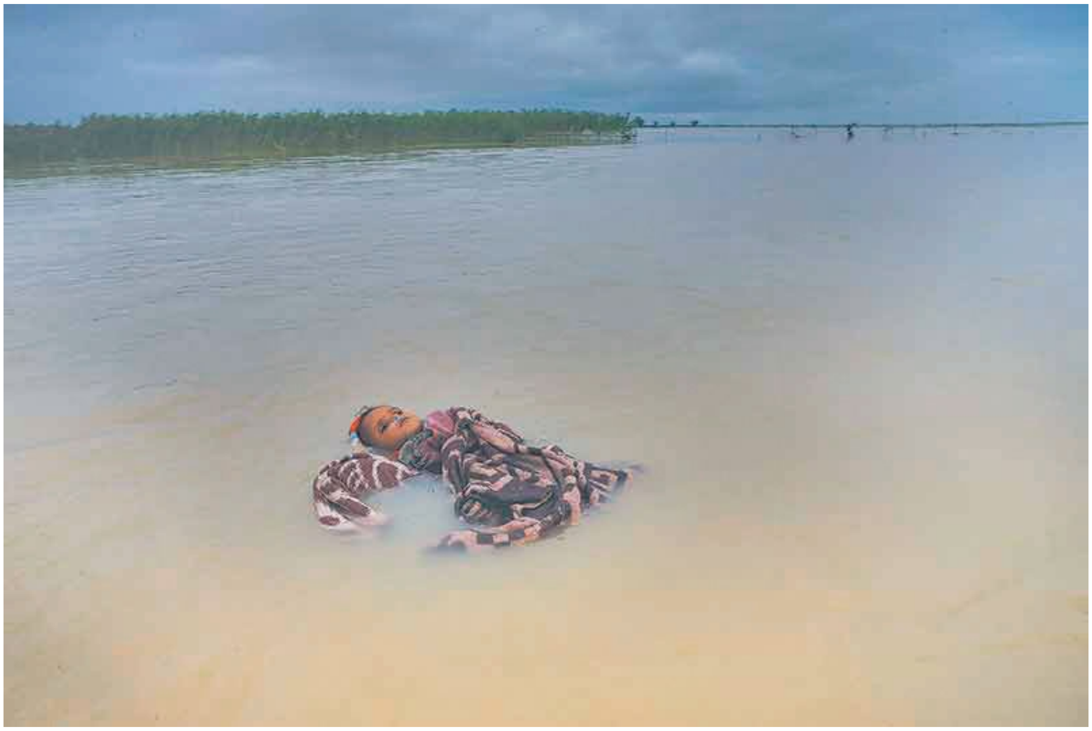

Project this photo.

A short presentation of the havoc caused by the flood in Terai in 2073.

Following this ask one of the girls to recite this poem.

कविता

बग्दै बग्दै जाने छोरासँग

विवश पोखरेल

भाद्र१७, २०७४

ढुङ्गो बनाएर एउटी आमाको मुटु
पानीमा बगाइदिएकी छु
मेरो खुसी / मेरो प्राण / मेरो सपना
जानू, बग्दैबग्दै जानू
जहाा माटो भेटिन्छ, त्यहीँ टक्क अडिनू
र समाधिस्थ हुनू
.
जीवन भरि माटो सँगै खेले पनि, माटो मै हुर्किए पनि
हामी सँग आफ्नो भन्ने
माटो नै कहाँ थियो र तँलाई गाड्नू ?
तैंले नाङ्गै भुतुङ्गै खेल्ने ऐलानी आँगन
सरकारको थियो
तेरो बाबुले बर्खामा जोतेर
हिउँदमा धानको सिला खोज्ने गरेको खेत
मालिकको थियो
हामी सँग माटो त त्यति मात्र थियो
जुन श्रमको पसिना सँगै
हाम्रा आङमा टाँस्सिएर आएका थिए

आज बाढी बनेर आएको आँसुका भेलमा
ती माटोलाई पनि खलल्ल पखालेर
अचानोमा राखेर आफ्नो मुटुतालाई बिदा गर्दैछु
जानू खुसीले जानू
जहाँ माटो भेटिन्छ त्यहीँ टक्क अडिनू
र समाधिस्थ हुनू ।

अनियन्त्रित छाल भएर आइरहेछन्
ता जन्मनु अघि र पछिका सम्झनाहरु
ता जन्मिएको दिन नेपाल बन्द थियो
अस्पताल बन्द थियो
हड्तालमा थिए डाक्टरहरु
एक हप्ता देखिका मन पाएर
सुत्केरी उकास्न नसक्नुको पीडामा
झोक्राइरहेको तेरो बाबु
थचक्क घर छेउको खेतको आलीमा बसेर
क्वााक्वाा रोइरहेको थियो
र भगवान्भनाउादो ढुङ्गो सँग
तेरो सुखद आगमन र मेरो सुस्वास्थ्यको
प्रार्थना गरिरहेको थियो
घाम र पानी दुवैले पोल्ने रूझाउने
घर नामको एउटा सानो टहरो मा ता जन्मिएको थिइस्
त्यहीं मालिकको घरको
उब्रिएकोजुठो र बासी भात खाएर
उक्सिएकी थिए म सुत्केरी
सुत्केरी भएको दिनमात्र एक दिन चिसो कुनामा
एक मुठी सन्ठी र एक मुठा पराल बालेर
सेकाएकी थिएँ आफ्नो चिसिएको आङ
तातो पानीलाई तेल बनाएर दल्दै
तङ्ग्राएकी थिएँ आफ्नो शरीर

तँलाई जीवनको गहिरो नदीमा
डुबुल्की मार्न सिकाउँदा सिकाउँदै
तैरिन सिकाउँदा सिकाउँदै
आफ्ना इन्द्रेनी सपना डुबाएर स्तब्धछु
खुसी डुबाएर स्तब्ध छु
ता यो देशमा दु:खै पाउन त आएको थिइस्
दु:खै पाएर गइस्
जानू....., तैंले पानी मा खेलेका कागज का नाउ जस्तै
कतै न डुबी बग्दै बग्दै जानू
अदृश्य बग्ने बतास र समय जस्तै सुस्तरी बग्दै बग्दै जानू
तालाई बेरेर बगाउन पनि म सँग
यो लाएको झुत्रो साडी बाहेक केही थिएन
जुन साडीको रङ
कुनै राजनैतिक दलको झन्डा सँग मिल्दैन
हाम्रा पसिना, आासु र जीवनसँग मिल्दैन
तथाकथित समानता, आरक्षण र स्वतन्त्रतासँग मिल्दैन
त्यही साडीलाई च्यातेर
तेरो अगाध मायालाई टपक्क निकाले र मुटुबाट
यो धमिलो पानीमा बगाइदिएकी छु
मेरो खुसी / मेरो प्राण / मेरो सपना
जानू, बग्दै बग्दै जानू
जहाा माटो भेटिन्छ त्यहीँ टक्क अडिनू
र समाधिस्थ हुनू ।

Exercise

Issues : Life of poor in Nepal, injustice, disparity.
Ask how they felt after listening to this poem.
Get a reaction from few students. (three-four students)
What are the issues arising from this poem? (only points)
Organize in several domains.
Divide into groups and get them to discuss.
Bring to plenary.
Conclusion.

Session V

Ask one of the students to read the poem in the class.

This is written by one of our students and won the first prize in a poem competition (2074)

"जिन्दगीको जन्म "

मन बहादुर दर्जी

प्रिय जिन्दगी !
म व्यग्र छु तिम्रो जन्म हेर्न
त्यो जन्म
जुन एक काल खण्ड पश्चात
देह भष्म पश्चात
शून्यताको चितामा
चिर निन्द्रमा कात्रो ओडछ
विलिन हुन
अर्को अस्तित्वमा लीन हुन
म आकुल छु
व्याकुल छु
जिन्दगीको जन्म हेर्न ।

यदि तिमी सभ्यताको जननी हौ भने,
आदिम अग्नी हौ भने
म स्वयं तिमीमा छु
एक आरोपित रुपमा, भ्रुणको स्वरुपमा
गर्भ गृहको अन्धकारमा
म अंकुरीत छु ।
म समाधीको स्वैरै कल्पित नाम हुँ या
पतित् पतिबाट घट्ने घटित घटनाको परिणाम
म जो सुकै किन नहुनु
म आफैँमा निर्माण हुँ
चलायमान अस्तित्वको
म निर्माण हु
लाखौमा रोजिएको एक को
प्रिय जिन्दगी
मेरो क्रियाशिलता दोसाँधमा छ
जीवन र मृत्युको
सायद म
मनव र दानव निर्मित
वैवाहिक सम्वन्ध अगाडिको
नाजायज हुँ प्राकृतिक समागमको
सायद म
नियमित सुहागरात पछिको
आसा हुँ
आगमनको
म जो कोही किन नहुँ
यदि मेरो 'हुनु'
गर्भधारणले,
चलायमान मांसपिण्डले
सम्भावनाले
समयकालखण्डले दिन्छ भने
तिमी मलाई अवसर दिन मान्दैनौ

किन मान्छौ
अनियमितताको वोझ
किन गर्छौ
नवजात अस्तित्वको गर्भपतन रोज
जवकी म आउदैमा हुँ
तिम्रो खोज
त्यसैले जिन्दगी‘म अग्रसर छु .....आफ्नै जन्म हेर्न
आफ्नै अस्तित्वको पहिलो श्वास फेर्न ।

Exercise

Issues: abortion – is it ethical? When might it be justified? View of the child to be ? view of the mother to be? What if you are asked to perform abortion within the legal framework, how will you feel?

Ask how they feel about the poem. (3-4 students)

Divide into groups and get them to discuss.

Bring to plenary.

Conclusion.
